# Entertainment at Hon. Geo. H. Pendleton’s

**Published:** 1867-06

**Authors:** 


ENTERTAINMENT AT HON. GEO. H. PENDLETON'S.

At three o'clock this afternoon the members of the Association, accompanied by their ladies, such as were here, took a ride to the suburban residence of Hon. Geo. H. Pendleton, by invitation of the Committee, who furnished carriages for the purpose. The party numbered about three hundred, and were agreeably entertained.

THIRD DAY'S PROCEEDINGS.

The American Medical Association met again Thursday morning, the hall being full, though not crowded as it was on Wednesday. President Askew called the meeting to order.

Dr. D. H. Storer arose to a question of privilege, namely the honor of the Association. It was in debt, and its first duty was to take measures to relieve its executive officers from their embarrassments. He moved that every member be assessed a tax of $2, to raise the necessary funds.

On motion, he was requested to prepare a subscription paper, and lay upon the table for voluntary subscriptions.

